# Unique fine scale village spatial-temporal distributions of *Anopheles farauti* differ by physiological state and sex

**DOI:** 10.1186/s13071-019-3815-y

**Published:** 2019-11-26

**Authors:** Edgar J. M. Pollard, Tanya L. Russell, Allan Apairamo, Thomas R. Burkot

**Affiliations:** 1 Australian Institute of Tropical Health and Medicine, James Cook University, Cairns, QLD 4870 Australia; 2 National Vector Borne Disease Control Program, Honiara, Solomon Islands

**Keywords:** *Anopheles farauti*, Barrier Screen, Solomon Islands, Males, Sugar-feds

## Abstract

**Background:**

The ecology of many mosquitoes, including *Anopheles farauti*, the dominant malaria vector in the southwest Pacific including the Solomon Islands, remains inadequately understood. Studies to map fine scale vector distributions are biased when trapping techniques use lures that will influence the natural movements of mosquitoes by attracting them to traps. However, passive collection methods allow the detailed natural distributions of vector populations by sex and physiological states to be revealed.

**Methods:**

The barrier screen, a passive mosquito collection method along with human landing catches were used to record *An. farauti* distributions over time and space in two Solomon Island villages from May 2016 to July 2017.

**Results:**

Temporal and spatial distributions of over 15,000 mosquitoes, including males as well as unfed, host seeking, blood-fed, non-blood fed and gravid females were mapped. These spatial and temporal patterns varied by species, sex and physiological state. Sugar-fed *An. farauti* were mostly collected between 10–20 m away from houses with peak activity from 18:00 to 19:00 h. Male *An. farauti* were mostly collected greater than 20 m from houses with peak activity from 19:00 to 20:00 h.

**Conclusions:**

*Anopheles farauti* subpopulations, as defined by physiological state and sex, are heterogeneously distributed in Solomon Island villages. Understanding the basis for these observed heterogeneities will lead to more accurate surveillance of mosquitoes and will enable spatial targeting of interventions for greater efficiency and effectiveness of vector control.
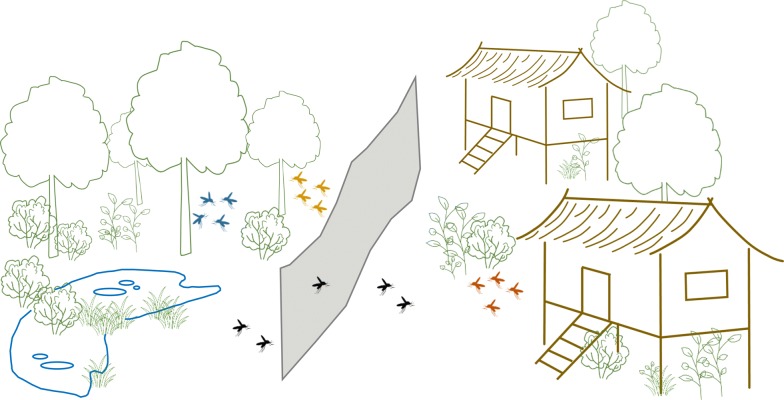

## Background

Mosquito ecology remains inadequately understood for many species [1, 2], including *Anopheles farauti*, a dominant malaria vector in the southwest Pacific from western Indonesia through Papua New Guinea and the Solomon Islands to Vanuatu [3, 4]. Although there are behavioural differences among species [5], in general, mosquitoes fly to satisfy five basic behaviours: to blood feed, to find favourable resting sites, to lay eggs, to mate and to sugar feed [2]. Much is known about the blood-feeding of *An. farauti* [6, 7] but less is known about resting [6, 8–10] and oviposition behaviours [11–13]. These behaviours directly impact the efficacy of the three WHO recommended interventions of insecticide-treated nets (ITNs), indoor residual spray (IRS) and larval source management (LSM) [14]. Further, very little is known about two behaviours of *An. farauti*, sugar-feeding and mating, both of which are targets of novel vector control tools. There are no published data on where or on what plants *An. farauti* prefer to take sugar meals and *An. farauti* swarms have also not yet been observed.

There are significant variations in activity patterns among species and these patterns are changing as mosquitoes respond differently to selection pressures induced by vector control and changing environmental conditions [15, 16]. Prior to IRS with DDT, *An. farauti* sought blood meals throughout the night, both indoors and outdoors. After the malaria elimination campaigns using IRS with DDT, a shift to earlier and more outdoor blood-feeding occurred. This behavioural shift was reinforced by the widespread deployment of ITNs to the point where 76% of biting now occurs outdoors before 21:00 h [3].

Knowledge of mosquito behaviours has been dominated by the use of traps with lures (including the use of humans and animals as baits) to define densities and distributions of species. Data generated in most traps provide useful “snapshots” on numbers of mosquitoes in specific physiological states, but these numbers may be biased by the lures used with traps. Thus, such data provides only limited insights into mosquitoes transitioning from one state to the next or where these behaviours take place (as lures induce mosquitoes to move towards the traps) [17]. There is a need to track mosquito behaviours without influencing the behaviours themselves to understand how best to monitor and control vector populations.

The barrier screen is an insecticide-free neutral (no bait or lure) net “trap” that intercepts mosquitoes as they fly in pursuit of blood meals, resting and oviposition sites, mating sites or sugar sources [18, 19]. Mosquitoes when flying between blood-feeding, oviposition, mating, sugar-feeding and resting sites temporarily stop to rest when encountering a barrier screen, from which they can be collected. This approach is advantageous to studies of mosquito distributions in that it does not alter the natural locations of mosquitoes with lures and it samples both male and female mosquitoes of all physiological states. The natural outdoor temporal and spatial distributions of *An. farauti* subpopulations by sex and physiological status were mapped within villages in the Solomon Islands using barrier screens.

## Methods

### Study sites

**Fig. 1:**
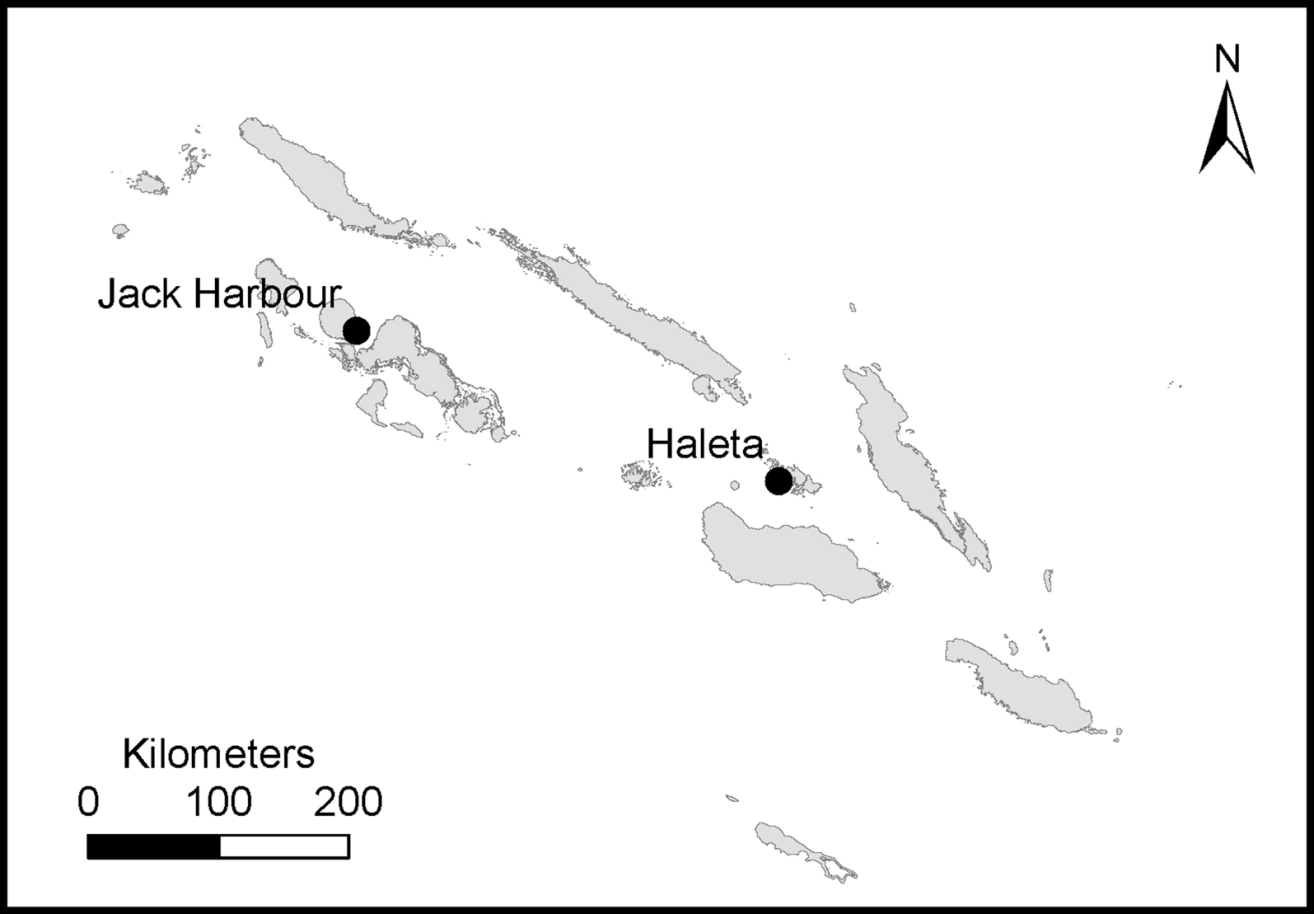
Map of Solomon Islands and showing Haleta village on Nggela Sule Island in Central Province and Jack Harbour village on Kolombangara Island in Western Province

The study was conducted in Jack Harbour village on Kolombangara Island in Western Province (8.059°S, 157.196°E) and Haleta village on Ngella Sule Island in Central Province (9.098°S, 160.115°E) in the Solomon Islands (Fig. 1) [20]. Both coastal villages are on mountainous, rain-forested islands; with a mean daily temperature of 27 °C and annual rainfall between 3000–5000 mm [21]. Haleta had a population of 366 in 70 households and Jack Harbour had a population of 151 in 38 households. Central Province had an annual parasite incidence (API) of 280 malaria cases per 1000 persons while Western Province had an API rate of 30 malaria cases per 1000 persons in 2016 [22].

*Anopheles farauti*, the dominant malaria vector in the Solomon Islands, is the only human-biting anopheline found in both villages with a mean of 14.8 bites per person per half-night (b/p/h-n) in Haleta village for 2011–2014 [3] and 26.3 b/p/h-n in Jack Harbour for 2014–2016 [20].

### Sampling adult mosquitoes

During each sampling period, mosquitoes were sampled simultaneously over 4–6 sequential nights from 18:00 to 00:00 h using both barrier screens (BS) and human landing catch (HLC) in May, August and November 2016 and February 2017. In addition, Haleta was sampled in July 2017.

Barrier screens were constructed from 20 m long black high-density polyethylene shade cloth of 70 % shading (160 g/m^2^; Coolaroo^®^ Gale Pacific Ltd, Melbourne, Australia) [18, 19]. Mosquitoes resting on barrier screens were collected by mouth aspiration for 15 min every hour by collectors to whom insect repellent had been applied. For each mosquito, the time of collection by hour; the side of the barrier screen and resting height above the ground [using 3 broad categories of low (0–0.6 m), medium (0.6–1.2 m) and high (1.2–1. m)] were recorded. On any given night, 8 barrier screens were deployed across a village, and the distance of barrier screens to the nearest house and primary larval habitat measured. Barrier screens were relocated to sample a wide range of habitats/locations. Host-seeking females were also captured by HLC outdoors at 10 sites distributed throughout each village. The same locations were used for all HLC sampling efforts during all sampling periods as described previously [3, 20].

Captured anophelines were held by hour and collection station until identified to species by morphology [8], and categorised to sex (male or female) and physiological state at the field sites. Unfed, blood-fed and gravid mosquitoes were identified according to Detinova [23]; mosquitoes with a distended abdomen with a clear, likely sugar meal will hereafter be referred to as sugar-fed.

### Weather measurements

Weather Meters (Kestrel 4500) with wind vanes recorded the temperature, humidity and wind speed and direction at ground level and at 1.8 m above the ground nightly during collections.

### Statistical analysis

Generalised linear models (GLMs) with Gaussian distribution were used to analyse differences in (i) the temporal density of mosquitoes compared between physiological states; (ii) the distance from nearest house and physiological state; and (iii) the resting density of mosquitoes with average temperature, humidity and wind speed during collections. The significance of the interaction was analysed using a Chi-square test (ANOVA) that compared the fit of two nested Poisson GLM models. The effect of barrier screen height on resting female mosquito densities was analysed with a Generalized Linear Mixed Model (GLMM) with a negative binomial distribution and a random factor for the date (glmer.nb; package = *lme4*). Samples without any resting mosquitoes were removed from the analysis. Incorporating date as the random factor into the GLMM model accounted for natural fluctuations in mosquito densities observed while increasing the power of the model. This analysis was conducted using R statistical software (ver.3.1.2).

### Geospatial analysis

Vector foci (areas with higher than mean densities) were determined using FleXScan (v3.1.2), a spatial Poisson distribution model to identify aggregated clusters by identifying spatial windows with greater ratios of observed to expected cases (relative risk). A single cluster detection was based on a spatial matrix defined using triangular irregular networks created based on Delaunay Triangulation, with Euclidian distance, limited to 10 stations with *P* < 0.01. The FleXScan identified foci were then mapped in ArcMap 10.1 with a 10 m buffer.

## Results

**Fig. 2:**
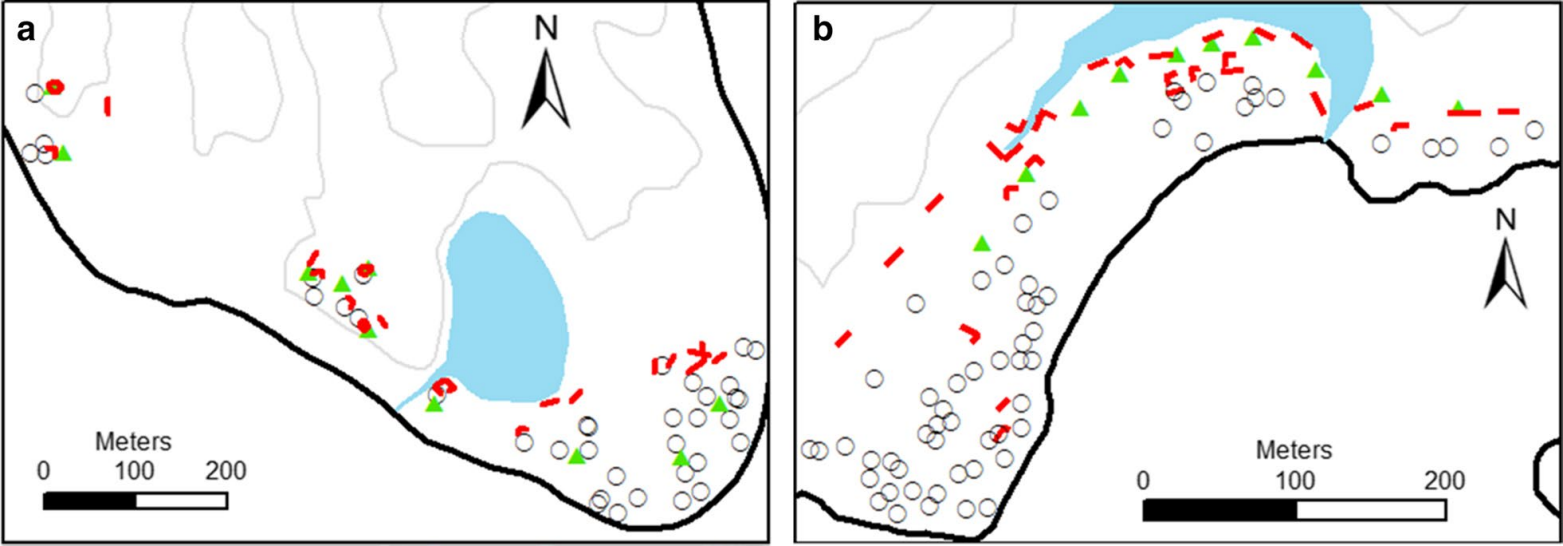
Village maps of Jack Harbour (**a**) and Haleta (**b**) showing all locations where barrier screens (red) and human landing catch stations (green) were located, as well as houses (circles) and primary larval habitat (blue)

A total of 3411 mosquitoes resting on barrier screens were collected: 2345 from Jack Harbour during 21 half nights and 1066 from Haleta during 28 half nights of collections. The positions of barrier screen positions in Haleta and Jack Harbour are shown in Fig. 2.

Of these, 2292 were *An. farauti* and 1283 were culicines. Ninety-two percent of the *An. farauti* collected were females (*n *= 2121) and 87% of the culicines were females (*n* = 1119). Culicines were composed of a mix of species in the genera *Culex* (*Cx. sitiens*, *Cx. quinquefasciatus*) and *Verallina* spp. There were also occasional rare collections of *Aedes* (*Ae. scutellaris*) and *Armigeres* spp. on the barrier screens. Of the female *An. farauti*, 67% were unfed (*n *= 1421), 23% blood-fed (*n *= 484), 8% sugar-fed (*n *= 173) and 2% gravid (*n *= 43). Mean number of resting female *An. farauti* per barrier screen per half-night (r/bs/h-n) during sampling periods ranged from 0.9 r/bs/h-n in the dry season to 11.0 r/bs/h-n in the wet season.

A total of 12,733 female, blood-seeking *An. farauti* were collected by HLC: 7296 from Jack Harbour (14 half nights) and 5437 from Haleta (20 half nights) villages over 34 half nights. Mean number of host-seeking female *An. farauti* per sampling period ranged from 1 b/p/h-n to 13 b/p/h-n).

### Mosquito distributions on barrier screens

**Fig. 3:**
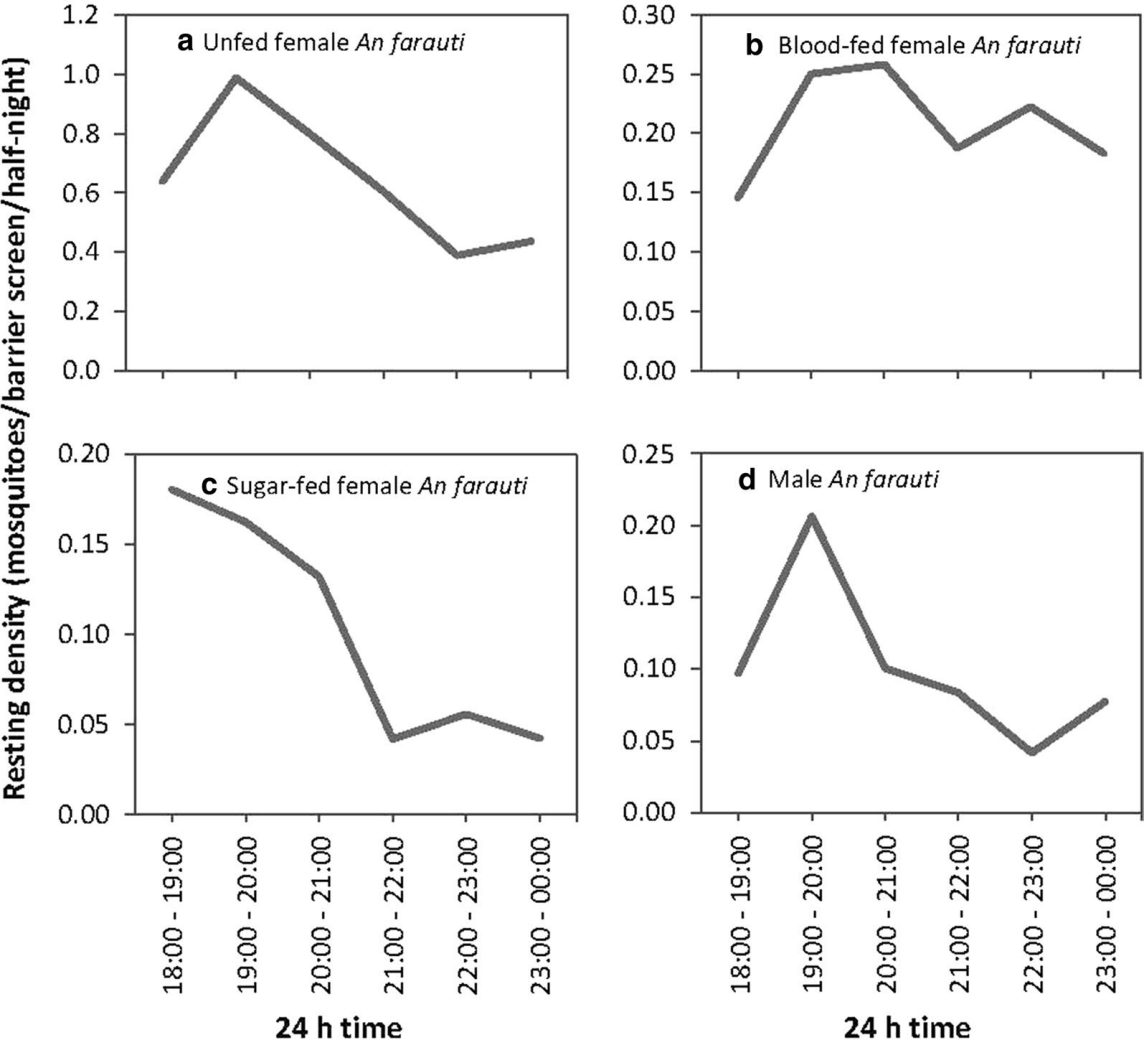
Densities of *An. farauti* on barrier screens by time. **a** Unfed females. **b** Blood-fed females. **c** Sugar-fed females. **d** Males

The hourly numbers of mosquitoes collected on barrier screens varied by physiological status (*χ*^2^ = −205.37, *df * = −36, *P* ≤ 0.0001). Numbers of unfed female and male *An. farauti* resting on the barrier screens peaked at 19:00–20:00 h, decreasing to 00:00 h when sampling ceased (Fig. 3). The number of blood-fed female *An. farauti* on barrier screens maintained a longer peak (from 19:00–21:00 h) and had a more gradual decline in numbers to 00:00 h. Sugar-fed female *An. farauti* were collected earlier in the evening, peaking at 18:00–19:00 h. Blood-seeking *An. farauti* females from HLC had a similar temporal patterns to resting unfed females on the barrier screens as also recorded in previous studies in the same villages [3].

There was a significant inverse association between the height above the ground where mosquitoes were collected and the mean numbers collected (β = −0.3596, SE = 0.1331, *P * = 0.007): 57% of *An. farauti* females were collected within 60 cm of the ground with a mean of 3.8 r/bs/h-n. In contrast, the mean resting density between 60 and 120 cm above the ground was 2.3 r/bs/h-n. Above 120 cm, only 1.2 r/bs/h-n *An. farauti* were collected.

### Geospatial resting locations

#### Distance from house

**Fig. 4:**
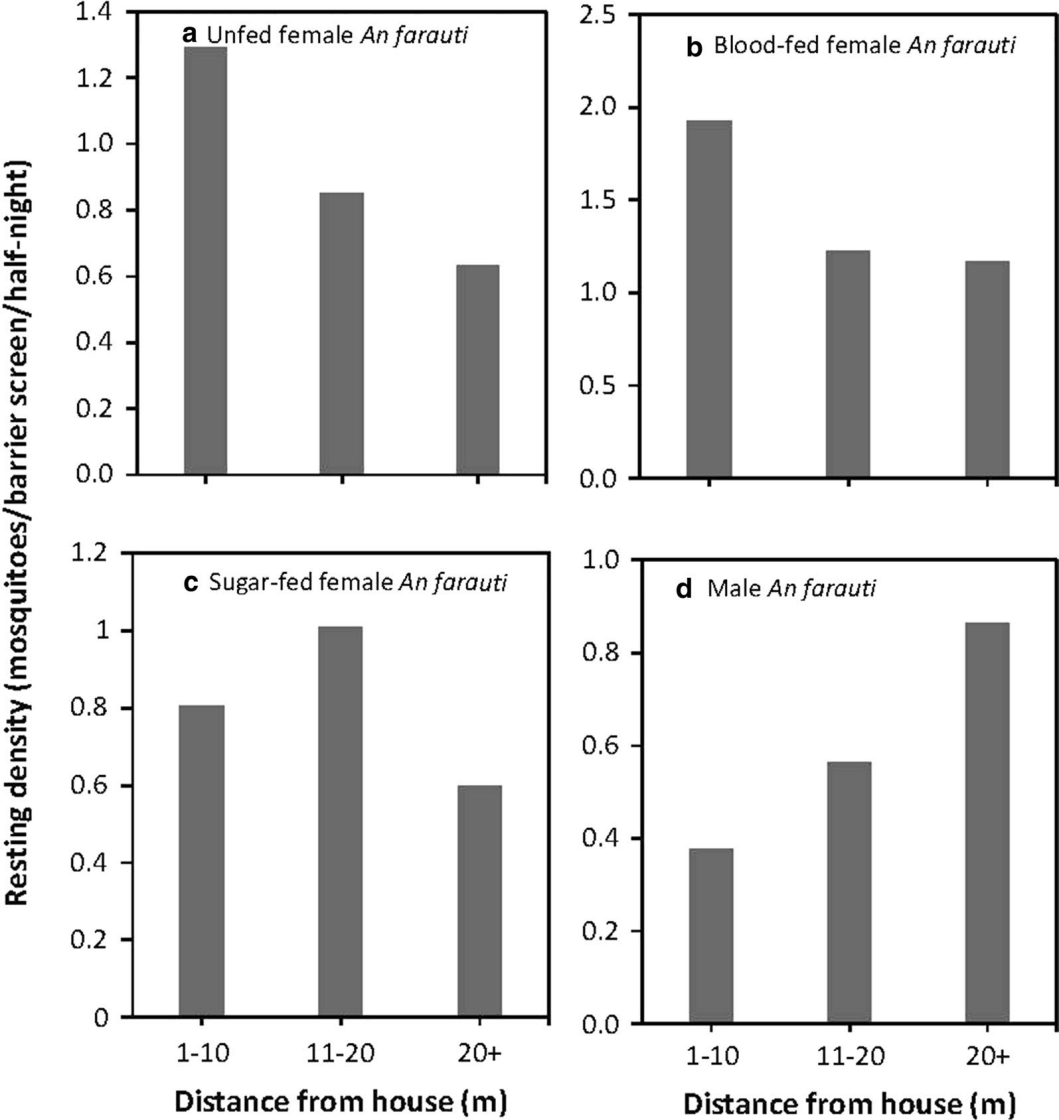
Densities of *An. farauti* on barrier screens in proximity to the closest house. **a** Unfed females. **b** Blood-fed females. **c** Sugar-fed females. **d** Males

There was a significant interaction between the distance to the house and the physiological state of resting female *An. farauti* (*χ*^2^ = −136.82, *df * = −4, *P* ≤ 0.001). Unfed and blood-fed *An. farauti* were most commonly collected within 10 m of a house, while more sugar-fed female *An. farauti* were collected 11–20 m from houses (Fig. 4). Male *An. farauti* numbers were highest at distances > 20 m from houses and also most (62%) were collected within 10 m of a large swamp especially evident in Haleta village.

#### Resting foci

**Fig. 5:**
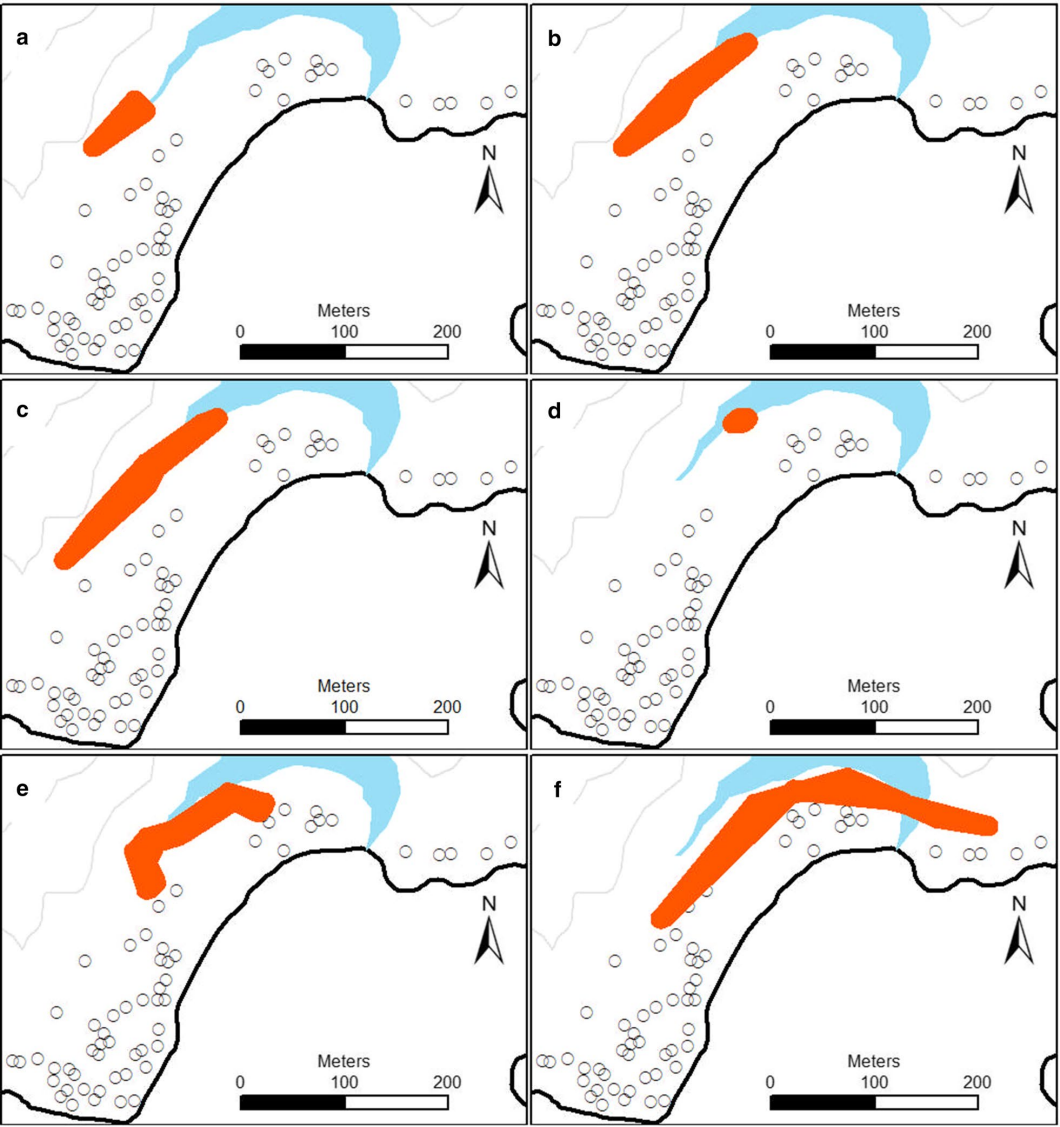
Locations of significant mosquito foci (shown in orange) in Haleta village, Central Province, Solomon Islands, collected by barrier screens (**a** unfed female *An. farauti*; **b** blood-fed female *An. farauti*; **c** sugar-fed female *An. farauti*; **d** male *An. farauti*; **e** female culicine species) and human landing catch (**f** blood-seeking female *An. farauti*)

**Fig. 6:**
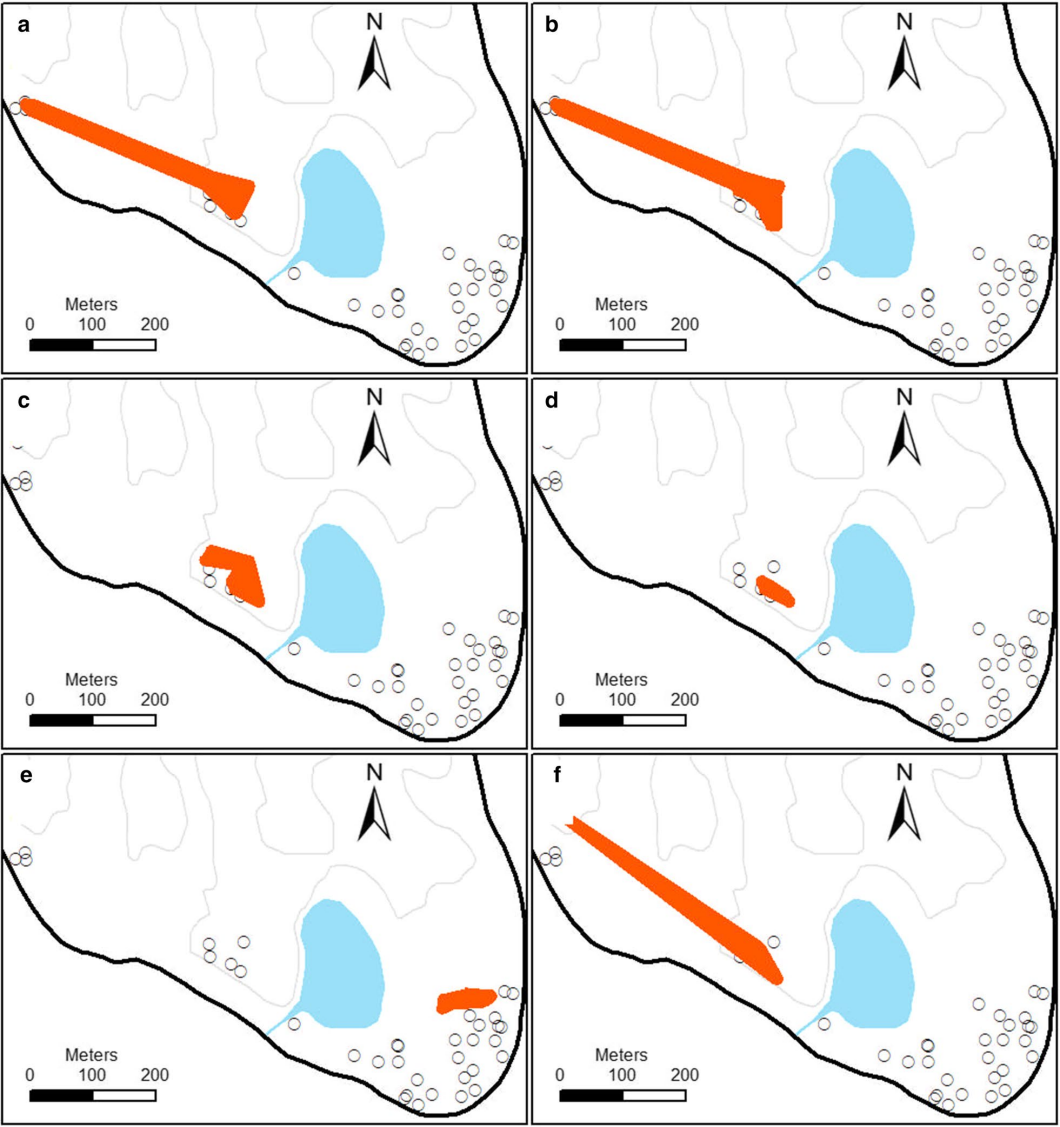
Locations of significant mosquito foci (shown in orange) in Jack Harbour village, Western Province, Solomon Islands, collected by barrier screens (**a** unfed female *An. farauti*, **b** blood-fed female *An. farauti*, **c** sugar-fed female *An. farauti*, **d** male *An. farauti*, and **e** female culicine species) and human landing catch (**f** blood-seeking female *An. farauti)*

Significant foci of mosquitoes by physiological states and species were identified within each village (Table 1). In Haleta and Jack Harbour villages, there was high spatial overlap where unfed female *An. farauti* and sugar-fed and blood-fed female *An. farauti* were collected (Figs. 5, 6). Sugar-*fed An. farauti* females and *An. farauti* males were also collected in close proximity, particularly evident in Jack Harbour. Blood-seeking *An. farauti* foci also tended to resemble patterns of unfed and blood-fed female *An. farauti.* Although there were differences between the villages, the male *An. farauti* foci was always smaller than the female foci. The populations of female culicines and anophelines (e.g. *An. farauti*) were largely segregated into different parts of the villages.

**Table 1:** Spatial clusters (foci) of *An. farauti* and *Culex* within Jack Harbour and Haleta villages

Species	Physiological state	Maximum distance (m)	Percent of locations (census areas)	Observed percent of mosquitoes	Expected no. of mosquitoes	Relative risk (obs/exp)
Haleta village						
*An. farauti*	Unfed female	52	7 (2/29)	28 (107/381)	30	3.61
*An. farauti*	Blood-fed female	141	14 (4/29)	33 (54/165)	31	1.75
*An. farauti*	Sugar-fed female	185	17 (5/29)	47 (28/60)	12	2.26
*An. farauti*	Male	0	3 (1/29)	39 (53/136)	11	4.74
*Culex* spp.	Female *Culex*	128	21 (6/29)	74 (211/287)	48	4.44
*An. farauti*	Blood-seeking female (HLC)	341	70 (7/10)	76 (4123/5437)	3806	1.08
Jack Harbour village						
*An. farauti*	Unfed female	368	18 (4/22)	59 (583/987)	223	2.61
*An. farauti*	Blood-fed female	385	32 (7/22)	61 (172/280)	99	1.75
*An. farauti*	Sugar-fed female	103	23 (5/22)	71 (80/113)	30	2.64
*An. farauti*	Male	40	9 (2/22)	70 (21/30)	4	4.90
*Culex* spp.	Female *Culex*	71	18 (4/22)	64 (525/824)	89	5.92
*An. farauti*	Blood-seeking female (HLC)	407	30 (3/10)	58 (4268/7296)	2189	1.95

*Note:* Foci were detected with a flexible scan statistic using FleXScan software and were significant at *P *< 0.05

#### Weather

**Fig. 7:**
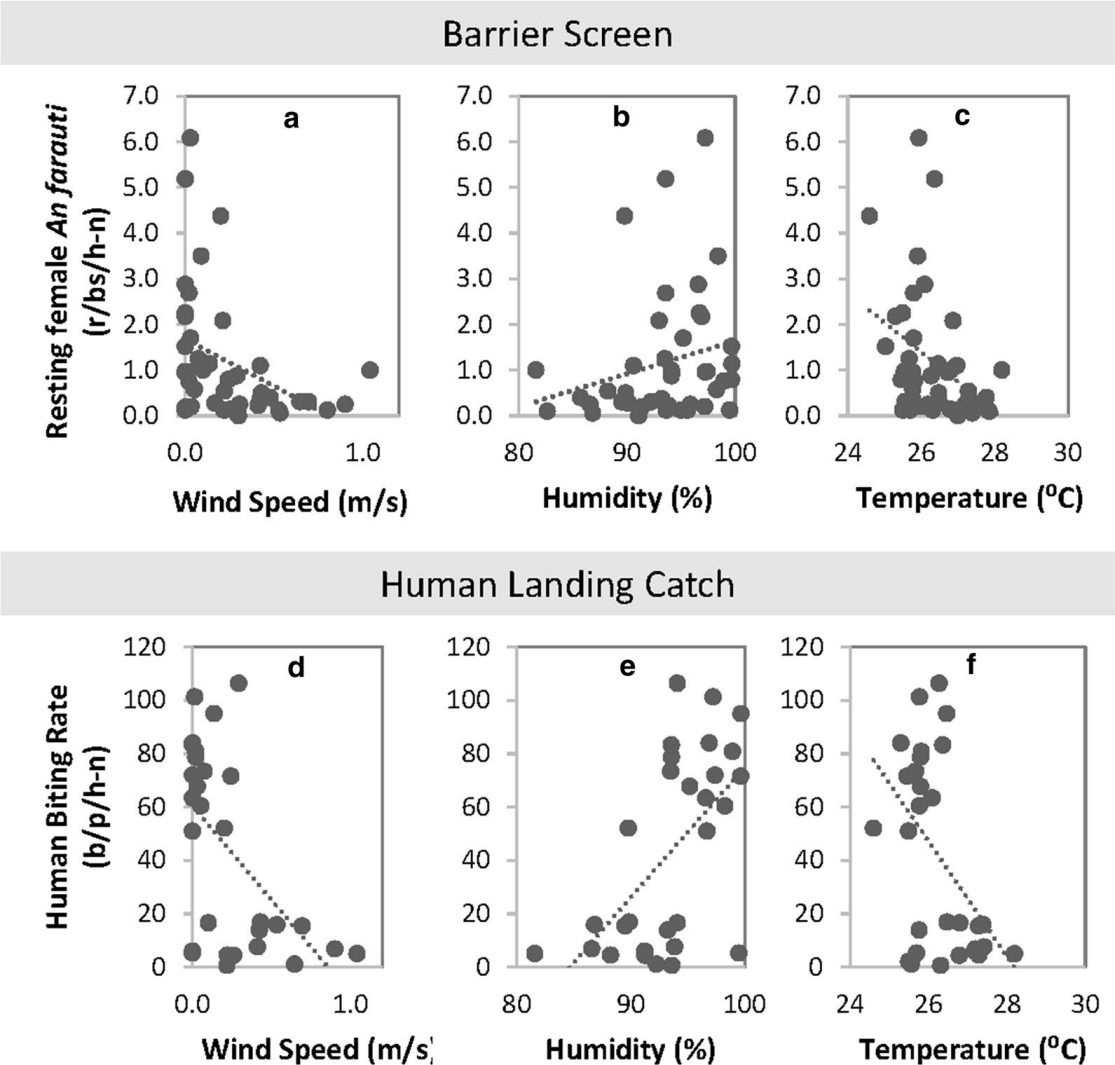
Relationships between weather parameters and average total female resting *An. farauti* per half night with linear trendline. **a** Average wind speed and average total female resting *An. farauti* per half night. **b** Average humidity and average total female resting *An. farauti* per half night. **c** Average temperature and average total female resting *An. farauti* per half night. **d** Average wind speed and average total female biting *An. farauti* per half night. **e** Average humidity and average total female biting *An. farauti* per half night. **f** Average temperature and average total female biting *An. farauti* per half night

Temperature, humidity and wind speed strongly influenced mosquito numbers on barrier screens (temperature: β= −0.4398, SE = 0.0340, *P* ≤ 0.001; humidity: β = −0.1570, SE = 0.0073, *P* ≤ 0.001; wind speed: β = −2.7890, SE = 0.1558, *P* ≤ 0.001) and with HLC (temperature: β = −0.2922, SE = 1.8985, *P* ≤ 0.001; humidity: β =  0.0516, SE = 0.0108, *P* ≤ 0.001; wind speed: β = −1.9914, SE = 0.2026, *P* ≤ 0.001). Higher average wind speeds were associated with lower *An. farauti* collections on barrier screens and with human landing catch (Fig. 7). Collection densities above 1 female *An. farauti* per night on barrier screens never occurred when average wind speeds were > 0.2 m/s. Lower average humidity during mosquito sampling was associated with lower numbers of *An. farauti* collected on barrier screens. Higher densities of female *An. farauti* on barrier screens were associated with lower average temperatures.

## Discussion

In villages, exposure to anophelines and malaria transmission is unevenly distributed [24]. Previous studies documented heterogeneity in the distribution of biting *An. farauti* in villages in the Solomon Islands and proposed that the risk of malaria was best estimated by biting rates in low-transmission villages [20]. This study expands our understanding of the distributions of mosquitoes to include other species, physiological states, sex and behaviours. Analogous to the temporal activity patterns of *An. farauti*, the spatial activity patterns for *An. farauti* in the Solomon Islands also differed by physiological states and sex. Whereas biting rates estimate the risk of malaria transmission, the distributions of other vectors by physiological states or behaviours may enable optimising control and monitoring strategies. This study also highlights the suitability of the barrier screen for collecting non-anopheline mosquitoes.

The complexity of the environment influences the locations where mosquitoes were sampled, with barrier screens near potential blood sources (houses) intercepting more unfed (potentially blood-meal seeking) and blood-fed *An. farauti*. The barrier screens were most efficient at sampling unfed mosquitoes (63–67%), followed by blood-fed (23–36%) with only 1–2% being gravid, as also seen from previous studies in PNG, Indonesia and the Solomon Islands [25, 26].

Here, the sugar-fed *An. farauti* were predominantly collected in the early evening, indicating that sugar-feeding is predominantly an early evening activity. This is similar to studies of the *An. gambiae* complex in Africa showing that sugar-feeding occurs early in the evening and morning (prior to blood-feeding) [27]. Generally, sugar-fed females and males, are collected but in low numbers (< 8%) on the barrier screens [25, 26], but with a high sampling effort sufficient numbers can be collected.

Across both villages, male *An. farauti* were collected in more geographically focused areas. Male *An. farauti* were mostly collected on barrier screens near larval habitats shortly after sundown suggesting that emergence of males or swarming occurs in the early evening. This is the first indication of possible times and locations of *An. farauti* mating as swarms in this species have yet to be documented.

Mosquito population dynamics is strongly impacted by weather [27–29]. Wind, temperature and humidity are major factors influencing mosquito flight [30]. Despite the limited range in temperature, humidity and wind speed recorded during the present study, significant impacts on the densities of resting and biting *An. farauti* were found: increased densities of biting and resting *An. farauti* were associated with higher humidities and lower temperatures (within the 24–30 °C range). The finding that wind speeds greater than 1 km/h can significantly reduce *An. farauti* flight is consistent with impact of wind on other species [31] and suggests that reductions in exposure to biting *An. farauti* can be obtained by avoiding protected areas in the early evening when most *An. farauti* bites occur.

Data from outdoor barrier screens and indoor resting behaviours suggest many anophelines fly and rest predominantly within a meter of the ground [19, 25]. The height above the ground where *An. farauti* were collected on barrier screens suggests that *An. farauti* predominantly flies within a metre of the ground in the Solomon Islands. These observations are consistent with the observation that bites from *An. farauti* in Papua New Guinea were significantly reduced even at elevations of 35 cm [32].

These data defining the heights at which *An. farauti* fly and the influence of wind on flight suggest that significant protection from biting *An. farauti* can be afforded by two simple human behaviours: avoidance of protected areas in the early evening to maximise wind exposure (and thereby minimising mosquito bites) in the evening before sleeping and then sleeping in elevated houses under a LLIN. Spatial foci of *An. farauti* within the village were clearly different from culicine species and also differed in having different peak times of activities, signifying different ecological niches. The differing distributions of the culicines and anophelines suggests that interventions for controlling these different mosquitoes may require different distribution strategies.

This research provides fundamental bionomic information that can be utilised to support the optimisation of novel vector control tools. Attractive targeted sugar baits (ATSB) are an “attract and kill” strategy, where a highly attractive sugar lure is integrated into bait stations with an oral toxicant, usually garlic [33, 34]. In this study, foci of sugar-fed mosquitoes were mapped which could enable more effective placement of ATSBs. Insecticide treatment of barrier screens are an unproven technology that has the potential to kill mosquitoes who contact the screens, and warrants further research as a possible vector control tool.

## Conclusions

*Anopheles farauti* subpopulations, as defined by physiological state and sex, were found to be heterogeneously distributed in Solomon Island villages. This heterogeneity is proposed to be a function of proximity to blood and sugar sources, as well as resting and oviposition sites with the density of mosquitoes at any given location moderated by weather parameters (temperature, humidity and wind). Understanding the basis for mosquito heterogeneities in villages will lead to more accurate surveillance of mosquitoes and greater efficiency and effectiveness of vector control tools. In the absence of new control tools, there are simple measures that individuals can take to protect themselves from mosquito bites based on an understanding of the factors that determine the distributions and densities of biting mosquitoes.

## Data Availability

The datasets supporting the conclusions of this article are available in the JCU Tropical Data Hub repository: http://doi.org/10.25903/5d4a446668e70.

## Abbreviations

API: annual parasite incidence
b/p/h-n: bites/person/half-night
BS: barrier screen
HBR: human-biting rate
HLC: human landing catch
EIR: entomological inoculation rate
IRS: indoor residual spraying
LLIN: long-lasting insecticidal net
r/bs/n: resting/barrier screen/half-night
